# Supplementary material for: Healthcare Costs and Resource Utilisation of Italian Metastatic Non-Small Cell Lung Cancer Patients
Source: Cancers (Basel). 2024 Jan 30;16(3):592. doi: 10.3390/cancers16030592 (PMC10854909; doi:10.3390/cancers16030592)
Supplement: Supplementary file 1 [file cancers-16-00592-s001.zip › cancers-2788209-supplementary.pdf]

Supplementary materials

Table S1. Second-line treatments administered by mutation status. The negative/unknown cohort was further grouped based on the availability of the 1L ICI monotherapy at 1L starting time (Pre-and Post-1L IO).

	**EGFR, ALK, or ROS1 Positive patients**	**EGFR, ALK, or ROS1 Negative/Unknown patients**	
**Second-line (2L) therapies**	**N=52 (%)**	**Pre-1L IO N=78 (%)**	**Post-1L IO N=111 (%)**
Multi-agents chemotherapy	7 (13.5)	11 (14.1)	12 (10.8)
Single-agent chemotherapy	6 (11.6)	38 (48.7)	16 (14.4)
Targeted therapy	37 (71.1)	24 (30.8)	—
Targeted therapy + chemotherapy	—	2 (2.6)	2 (1.8)
PD-1/PD-L1 inhibitor single agent	1 (1.9)	3 (3.8)	81 (73.0)
PD-1/PDL1 inhibitor + chemotherapy	1 (1.9)	—	—

Table S2: Third-line treatments administered by mutation status. The negative/unknown cohort was further grouped based on the availability of the 1L ICI monotherapy at 1L starting time (Pre-and Post-1L IO).

	**EGFR, ALK, or ROS1 Positive patients**	**EGFR, ALK, or ROS1 Negative/Unknown patients**	
**Third-line (3L) therapies**	**N=23 (%)**	**Pre-1L IO N=17 (%)**	**Post-1L IO N=32 (%)**
Multi-agents chemotherapy	5 (21.7)	3 (17.6)	4 (12.5)
Single-agent chemotherapy	4 (17.4)	7 (41.2)	24 (75.0)
Targeted therapy	12 (52.2)	5 (29.4)	—
Targeted therapy + chemotherapy	—	—	1 (3.1)
PD-1/PD-L1 inhibitor single agent	2 (8.7)	2 (11.8)	3 (9.4)
